# Whither the genus *Caldicellulosiruptor* and the order Thermoanaerobacterales: phylogeny, taxonomy, ecology, and phenotype

**DOI:** 10.3389/fmicb.2023.1212538

**Published:** 2023-08-03

**Authors:** Ryan G. Bing, Daniel J. Willard, James R. Crosby, Michael W. W. Adams, Robert M. Kelly

**Affiliations:** ^1^Department of Chemical and Biomolecular Engineering, North Carolina State University, Raleigh, NC, United States; ^2^Department of Biochemistry and Molecular Biology, University of Georgia, Athens, GA, United States

**Keywords:** *Caldicellulosiruptor*, Thermoanaerobacterales, bacteria, thermophiles, phylogeny, ecology, fermentative anaerobes

## Abstract

The order Thermoanaerobacterales currently consists of fermentative anaerobic bacteria, including the genus *Caldicellulosiruptor*. *Caldicellulosiruptor* are represented by thirteen species; all, but one, have closed genome sequences. Interest in these extreme thermophiles has been motivated not only by their high optimal growth temperatures (≥70°C), but also by their ability to hydrolyze polysaccharides including, for some species, both xylan and microcrystalline cellulose. *Caldicellulosiruptor* species have been isolated from geographically diverse thermal terrestrial environments located in New Zealand, China, Russia, Iceland and North America. Evidence of their presence in other terrestrial locations is apparent from metagenomic signatures, including volcanic ash in permafrost. Here, phylogeny and taxonomy of the genus *Caldicellulosiruptor* was re-examined in light of new genome sequences. Based on genome analysis of 15 strains, a new order, Caldicellulosiruptorales, is proposed containing the family *Caldicellulosiruptoraceae*, consisting of two genera, *Caldicellulosiruptor* and *Anaerocellum*. Furthermore, the order Thermoanaerobacterales also was re-assessed, using 91 genome-sequenced strains, and should now include the family Thermoanaerobacteraceae containing the genera *Thermoanaerobacter, Thermoanaerobacterium, Caldanaerobacter,* the family Caldanaerobiaceae containing the genus *Caldanaerobius*, and the family Calorimonaceae containing the genus *Calorimonas*. A main outcome of ANI/AAI analysis indicates the need to reclassify several previously designated species in the Thermoanaerobacterales and Caldicellulosiruptorales by condensing them into strains of single species. Comparative genomics of carbohydrate-active enzyme inventories suggested differentiating phenotypic features, even among strains of the same species, reflecting available nutrients and ecological roles in their native biotopes.

## 1. Introduction

Fermentative anaerobes are biotechnologically relevant microorganisms; the push for renewable energy has identified them as microbial platforms because of their ability of convert plant polysaccharides, including in some cases microcrystalline cellulose, into industrially important products, such as acetone, butanol and ethanol (Bing et al., 2022). In fact, bioprocesses, used to produce industrial solvents prior to the emergence of petrochemicals, have received renewed attention in recent years with the development of molecular genetic tools to facilitate metabolic engineering of fermentative anaerobes (Lee et al., 2012; Charubin et al., 2018).

The taxonomy of fermentative anaerobes (Wagner and Wiegel, 2008), a taxonomically heterogeneous group, is in flux, and not without some controversy (Robitzski, 2022). For example, re-naming of the phylum ‘Firmicutes’ to ‘Bacillota’, which includes fermentative anaerobes, was proposed to reflect the fact that it also contains aerobic bacteria from the genus *Bacillus* (Oren and Garrity, 2021). The wide availability of genome sequences has displaced 16S rRNA gene sequence as the primary standard for differentiating one microbe from another. This may ultimately lead to purely alphanumeric designations for microorganisms, replacing the binomial nomenclature devised by Linnaeus centuries ago that still serves as the basis for the International Code of Nomenclature of Prokaryotes (Akst, 2021). For now, genus-species designations remain the taxonomic standard, such classifications can now be assessed using whole genome sequence comparisons. Absent from this characterization, however, is phenotypic information that is central to discerning features that ultimately define the microbiology. Phenotype can map to small sections of the genome not apparent from phylogenetic analysis or taxonomy.

Within the phylum Bacillota (formerly Firmicutes) are many thermophilic fermentative anaerobes currently assigned to the order Thermoanaerobacterales. These bacteria are sources of thermostable enzymes (i.e., glycoside hydrolases) capable of hydrolyzing a wide range of plant polysaccharides (Blumer-Schuette et al., 2008, 2014; Conway et al., 2016, 2018) present in lignocellulosic biomass (Zeldes et al., 2015; Straub et al., 2017, 2019, 2020). This biotechnological perspective serves as motivation to re-visit the taxonomy and phylogeny of fermentative anaerobes currently assigned to the order Thermoanaerobacterales to assess whether current classifications are consistent with emerging genome sequence information. Of particular interest here is the placement of those extremely thermophilic Thermoanaerobacterales (T_opt_ > 70°C) that are currently all assigned to the genus *Caldicellulosiruptor* (Blumer-Schuette et al., 2010; Blumer-Schuette, 2020).

Table 1 lists the 13 species (and two strains) currently assigned to the genus *Caldicellulosiruptor* for which genome sequences are available, in addition to information about their genomes and isolation sites. Genomes average 2.71 ± 0.18 Mb with 2,604 ± 181 ORFs and G + C content of 35.6 ± 0.5%. Note that it was recently proposed that *Caldicellulosiruptor acetigenus* is actually composed of three previously described species (*C. acetigenus*, *C. kristjanssonii*, *C. lactoaceticus*; Habib et al., 2021) and should be re-classified into a single species and associated strains. Members of *Caldicellulosiruptor* are globally distributed (Blumer-Schuette, 2020). *Caldicellulosiruptor saccharolyticus* (f. *Caldocellum saccharolyticum*; Luthi et al., 1990; Gibbs et al., 1992), the first named species discovered, was isolated from a piece of wood downstream from a 78°C pool in Taupo, New Zealand, as were many other thermophilic isolates from similar sites in New Zealand that were not designated with taxonomic classifications at that time (Patel et al., 1986). From that group, *Caldicellulosiruptor danielii* and *Caldicellulosiruptor morganii* were eventually named when their genome sequences were reported (Lee et al., 2018). *Caldicellulosiruptor* species have since been isolated from globally diverse thermal features (e.g., Yellowstone National Park, United States; Hveragerði, Iceland; Nagano Prefecture, Japan; Kamchatka, Russia; Changbai, China) and, based on metagenomic data (Blumer-Schuette, 2020), yet uncharacterized species likely inhabit neutral, terrestrial, thermal features wherever they occur on earth. To date, *Caldicellulosiruptor bescii* (Kataeva et al., 2009; Yang et al., 2010; f. *Anaerocellum thermophilum*; Svetlichnyi et al., 1990) has been the focus of most studies of these bacteria, given the availability of molecular genetic tools (Chung et al., 2014; Lipscomb et al., 2016) and biotechnological potential (Lee et al., 2020; Bing et al., 2022) of this species. Not only has *C. bescii* been engineered to produce industrial chemicals (Williams-Rhaesa et al., 2018; Straub et al., 2020), but can also degrade and ferment transgenic lignocellulose (Straub et al., 2019). With the recent availability of additional complete genome sequences (Kataeva et al., 2009; Elkins et al., 2010; Lee et al., 2015; Mendoza and Blumer-Schuette, 2019; Habib et al., 2021; Bing et al., 2023b), it is essential that we re-visit the classification of the *Caldicellulosiruptor* and its taxonomic placement within the order Thermoanaerobacterales to give an updated phylogenetic perspective on these biotechnologically-important microorganisms.

**Table 1:** Isolation and genome sequence information of species currently assigned to genus *Caldicellulosiruptor.*

Species	Isolation site	Isolation environment	Genome (Mb)	ORFs	G + C (%)	Ref
*saccharolyticus*	Taupo, New Zealand	Wood in hot spring pool (48°C)	2.97	2,924	35.2	van de Werken et al. (2008)
*changbaiensis*	Changbai Mountains, China	Hot spring sediment (83°C, pH 7)	2.91	2,833	35.1	Mendoza and Blumer-Schuette (2019)
sp. str. F32	Qingdao, China	Biocompost	2.38*	2,426	35.2	Ying et al. (2013)
*naganoensis*	Nozawaonsen, Nagano Pref., Japan	Hot spring mud & sediment (75–85°C, pH 9.0)	2.49	2,473	35.3	Lee et al. (2015)
*morganii*	Rotorua, New Zealand	Hot spring (63°C, pH 8.8)	2.48	2,413	36.5	Lee et al. (2015)
*danielii*	Waimangu, New Zealand	Hot spring	2.83	2,714	35.8	Lee et al. (2015)
*hydrothermalis*	Pauzhetka, Kamchatka, Russia	Terrestrial neutral hot spring	2.77	2,679	36.1	Blumer-Schuette et al. (2011)
*diazotrophicus*	Nakabusa, Nagano Pref., Japan	Hot spring N_2_-poor biomats (78.3°C, pH 8.5–8.9)	2.60	2,449	34.8	Chen et al. (2021b)
*owensensis*	Owens Lake, CA, United States	Freshwater pond sediment in Dry Lake Bed (32°C, pH 9.0)	2.43	2,333	35.4	Blumer-Schuette et al. (2011)
*obsidiansis*	Yellowstone Nat. Park, WY, United States	Hot spring (66°C, pH 5.0)	2.53	2,404	35.2	Elkins et al. (2010)
*bescii*	Valley of Geysers, Kamchatka, Russia	Hot spring caused waterlogged foot of a geyser (90°C, pH 6.5)	2.93	2,828	35.2	Yang et al. (2010)
*kronotskyensis*	Valley of Geysers, Kamchatka, Russia	Terrestrial neutral hot spring	2.84	2,623	35.1	Blumer-Schuette et al. (2011)
*acetigenus (acetigenus)*	Hveragerði-Hengill, Iceland	Hot spring biomats and sediments (55–75°C, pH 8.0–8.7)	2.74	2,643	36.3	Onyenwoke et al. (2006) and Habib et al. (2021)
*acetigenus (kristjanssonii)*			2.80	2,712	36.0	
*acetigenus (lactoaceticus)*			2.67	2,601	36.1	

^*^Genome sequence not closed; 127 contigs.

## 2. Materials and methods

Unless noted otherwise, all computer software parameters used were default parameters.

### 2.1. Strain and genome sequence information

Genome assemblies for currently classified Thermoanaerobacterales (based on NCBI taxonomy, March 2023) were obtained from the National Center for Biotechnology Information (NCBI). The list of species strains and accession numbers are provided in Supplementary Table S1. 16S ribosomal RNA gene sequences were retrieved based on NCBI annotations for the most complete 16S rRNA sequence found in these assemblies.

Isolation location coordinates for the *Caldicellulosiruptor* were obtained primarily through isolation literature and NCBI biosample information. When needed, locations were estimated as ‘best possible’ from information contained in the literature (e.g., town names or geographical features). Supplementary Tables S2, S3 contain detailed information on the locations and their accuracy. Geographical distances between locations were calculated *via* a webserver employing the formula developed by Vincenty (1975).^1^

### 2.2. Phylogenetic tree inference: 16S ribosomal RNA and genome taxonomy database bac120

16S rRNA gene sequences were aligned using Clustal Omega (v. 1.2.4; Sievers et al., 2011) using flags “--outfmt fa --distmat-out --full --full-iter --percent-id --guidetree-out.” The percent identities output was used for 16S rRNA identity matrices (Supplementary Table S8), which were color coded based on proposed 16S rRNA taxonomic rank “Minimum Sequence ID” values (Yarza et al., 2014). The 16S rRNA alignment was inputted into FastTree (v. 2.1.11; Price et al., 2010) to generate a distance tree using flags “-nt -gtr -gamma.” The 16S rRNA tree was mid-point rooted with Dendroscope 3 (Huson and Scornavacca, 2012).

The bac120 gene marker set from the Genome Taxonomy Database (GTDB) was also used to infer a phylogenetic tree from the same set of Thermoanaerobacterales (based on NCBI taxonomy, March 2023) using GTDB-Tk v2.1.0 (Parks et al., 2018, 2020, 2021; Chaumeil et al., 2019, 2022). The phylogenetic tree was inferred using the “de_novo_wf” workflow with 200 aa per gene marker and using the phylum Thermodesulfobiota as the out-group for tree rooting. The GTDB reference database was excluded to allow for a custom taxonomic classification. Both the 16S rRNA and bac120 trees were visualized and formatted using the Interactive Tree of Life (iTOL; Letunic and Bork, 2006). A color-blind friendly divergent color palette was applied to the different genera (determined by Chroma.js Color Palette Helper)^2^ (colors: #1d1b73, #243694, #2b53af, #2d74be, #009ba7, #b1ab14, #ba971c, #c5801d, #d36219, #f00000). The same genera colors were applied to the 16S rRNA tree.

### 2.3. Average identity calculations and pangenome analysis

Average nucleotide identities (ANI) were generated with pyani (v. 0.2.12; Pritchard et al., 2016) average_nucleotide_identity.py BLAST+ method (ANIb; flag “-m ANIb”). Final bidirectional ANIb values were generated by averaging the ANIb values representing the two orientations of the same strain pairs (Supplementary Table S6). Average amino acid identities (AAI) were calculated with GET_HOMOLOGUES (v22082022; Contreras-Moreira and Vinuesa, 2013; Vinuesa and Contreras-Moreira, 2015) with flags “-A –t 0 –M (Supplementary Table S7).” A composite ANIb/AAI matrix of the 91 genomes was calculated and heat-mapped based on the min/max values (40.4–100%) using the Inferno colormap. This same colormap scale was maintained for all ANIb/AAI figures.

Core and pangenome analysis were also completed using GET_HOMOLOGUES. For this, the flags “-A –t 0 –c –z –P –M” were used. Then compare_clusters.pl. was used to generate the core, soft core, and pangenomes. Finally, parse_pangenome_matrix.pl. with “-f core_Tettelin” was used to generate pan/core genome graphs (Supplementary Data S2).

### 2.4. Analysis of carbohydrate active enzymes (CAZymes)

For annotation of carbohydrate active enzymes, dbCAN (v 3.0.7; Zhang et al., 2018; Zheng et al., 2023) was locally run with the run_dbcan command using protein.faa files of translated nucleotide coding sequences as input and flags “--signalP = true --gram P” to use SignalP v 4.1 (Nielsen, 2017; Supplementary Data S2). The dbCAN output was then curated where all glycosyl transferases (GTs) were removed, and a consensus was generated between the three prediction algorithms (HMMER, eCAMI, and DIAMOND), where annotations that had agreement between at least 2 prediction algorithms was used as the consensus. Any proteins that had positive hits on only a single algorithm were subjected to a BLAST search on the NCBI webserver against the NCBI non-redundant protein sequences database to obtain an annotation. Any coding sequence deemed to not be involved in carbohydrate catabolism, based on the BLAST search annotation, were removed from the analysis, along with any coding sequences corresponding to proteins under 100 amino acids (Supplementary Table S9). For the Caldicellulosiruptorales, remaining proteins representing the “CAZysome” of each strain were input into GET_HOMOLOGUES for Pan/Core/Soft-core analysis, as described above for whole genomes. The resulting Core/Soft-cores of the Caldicellulosiruptorales were then analyzed for CAZyme annotation and predicted function (Supplementary Table S4).

### 2.5. Substrate use evaluation of *Caldicellulosiruptor diazotrophicus*

Three strains of Caldicellulosiruptorales were obtained from DSMZ-German Collection of Microorganisms and Cell Cultures GmbH: DSM 6725 (*C. bescii*), DSM 18901 (*C. hydrothermalis*), and DSM 112098 (*C. diazotrophicus*). Strains were adapted to substrates and grown in 125 mL serum bottles, as described previously (Bing et al., 2023a) where modified D671 media with cellobiose (Biosynth-Carbosynth, OC04040) was used for routine growth. All cultures were grown at 75°C with 150 rpm shaking in a New Brunswick Innova 42 incubator shaker. Adaption to beechwood xylan (Biosynth-Carbosynth, YX45751), wet-milled corn fiber (WMCF, provided by Novozymes A/S), or Avicel PH-101 (Millipore-Sigma) was done by passages of 5 × 10^8^ cells (final 1 × 10^7^ cells/ml starting cell density) from 5 g/L cellobiose cultures to 0.5 g/L cellobiose and 4.5 g/L carbohydrate equivalent of substrate (xylan, WMCF, or Avicel). Cell growth was monitored by epifluorescence microscopy, as previously described (Bing et al., 2023a). Once cells reached 5 × 10^8^ cells/ml or after 3 days, 5 × 10^8^ cells were passaged to 5 g/L carbohydrate equivalent of substrate alone. Culture growth was monitored for 7 days. *C. bescii* (DSM 6725) and *C. hydrothermalis* (DSM 18901) were included as controls for expected growth phenotypes on the substrates. All strains were expected to grow on cellobiose, xylan, and WMCF, but only *C. bescii* grows on microcrystalline cellulose (Avicel). The phenotype observed is robust growth of *C. bescii* on all substrate adaptions and final passages; *C. hydrothermalis* had robust growth on cultures containing cellobiose, xylan, and WMCF, weak growth on the adaption passage to Avicel (containing 0.5 g/L cellobiose) and no growth on Avicel alone.

## 3. Results

### 3.1. Proposed changes to the classification of the Thermoanaerobacterales and the genus *Caldicellulosiruptor*

**Figure 1:**
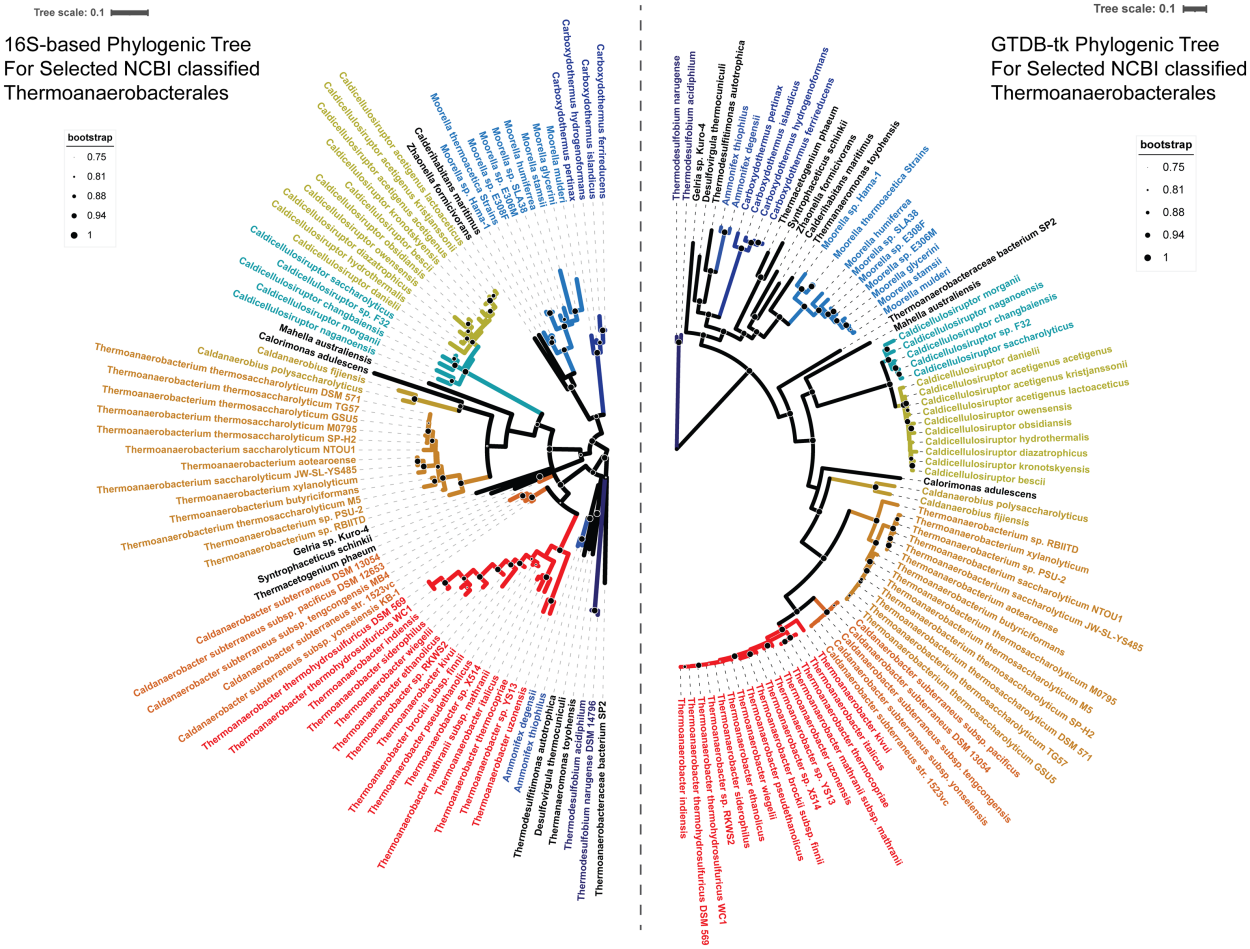
Phylogenic trees of currently classified Thermoanaerobacterales based on 16S rRNA sequence identity and Genome Taxonomy Database (GTDB-tk) analysis. Left tree structure was generated by FastTree from a CLUSTAL-Omega alignment of 16S ribosomal RNA sequences. The 16S rRNA was subsequently midpoint rooted with Dendroscope 3. Right tree structure was generated using 24,000 amino acid alignments by GTDB-tk with the ‘bac120’ gene markers from the Genome Taxonomy Database (GTDB, release 207). Bootstrap values between 75 and 100% are depicted with black circles with white outlines with, scaled as indicated by the legends. All other bootstrap values <75% are not shown. A color-blind friendly divergent color palette was applied to the GTDB tree to indicate members of the same genus; 16S tree nodes were colored to match the GTDB color pattern. *Caldicellulosiruptor* was split in two colors to show the proposed division into *Anaerocellum* and *Caldicellulosiruptor*.

In order to assess the existing taxonomic classification for the genus *Caldicellulosiruptor,* phylogenetic trees were inferred for a set of selected genomes within the order Thermoanaerobacterales (according to NCBI Taxonomy as of March 2023) using both 16S ribosomal RNA gene sequences to represent the existing taxonomic structure and the bac120 gene markers from the Genome Taxonomy Database (GTDB) to assess reclassification (Figure 1). The larger bac120 gene marker set allowed a multiple sequence alignment of ~24,000 amino acids, enabling more robust placement of the selected genomes within the tree and highlights the several areas needed for reclassification. Note that recent publications have already moved *Thermodesulfobium* to its own phylum Thermodesulfobiota (Frolov et al., 2023) and the genera *Calderihabitans*, *Desulfitibacter*, *Moorella*, and *Zhaonella* to the novel order Moorellales (Lv et al., 2020); however, these changes were not yet reflected in the NCBI taxonomy at the time of this analysis.

The 16S ribosomal RNA gene sequence tree includes multiple nodes at the genus level and higher with bootstrap values ≤0.50, indicating low confidence some of the taxonomic arrangements in this tree. Meanwhile, the bac120 gene marker tree has ≥0.50 bootstrap values (and ≥ 0.98 with the exception of the *Calorimonas adulescens* node) for all nodes above the species level, implying strong phylogenetic relationships above species-level classification. Based on the bac120 gene marker phylogeny, the Order Thermoanaerobacterales should consist of the Family Thermoanaaerobacteraceae containing the genera *Thermoanaerobacter*, *Thermoanaerobacterium*, and *Caldanaerobacter*, the Family Caldanaerobieaceae containing the genus *Caldanaerobius*, and the Family Calorimonaceae containing the genus *Calorimonas*. The current genus *Caldicellulosiruptor* is sufficiently divergent from the order Thermoanaerobacterales that it should be long to a separate Order, Caldicellulosiruptorales, and Family, Caldicelluosiruptoraceae. Within the Family, the species *C. danielli*, *C. hydrothermalis, C. diazotrophicus, C. owensensis, C. obsidiansis, C. bescii, C. kronotskyensis, C. acetigenus, C. lactoaceticus,* and *C. kristjanssonii* should be moved to a new genus; we propose this genus be named “*Anaerocellum”* in view of the fact that the type strain *C. bescii* was originally named *Anaerocellum thermophilum* (Svetlichnyi et al., 1990; Kataeva et al., 2009; Yang et al., 2010). The species *C. changbaiensis, C.* sp. F32, *C. saccharoloyticus, C. naganoensis,* and *C. morganii* should remain in the genus *Caldicellulosiruptor,* recognizing that the type strain from this group was *C. saccharolyticus* (Rainey et al., 1994).

Together, the orders Thermoanaerobacterales and Caldicellulosiruptorales likely comprise their own Class (Thermoanaerobacteria), but without comprehensive evaluation of the Class Clostridia, this could not be fully established here (Supplementary Table S5). The placement of *Mahella australiensis* in the bac120 gene marker tree further supports the need for a broader evaluation of the Class Clostridia. The GTDB taxonomy (~5,000 amino acid alignment) keeps the genus *Mahella* within Clostridia, but our ~24,000 amino acid alignment suggests that *Mahella* is more closely related to the Caldicellulosiruporales, meriting a Family-level classification within the Order Caldicellulosiruptorales.

**Figure 2:**
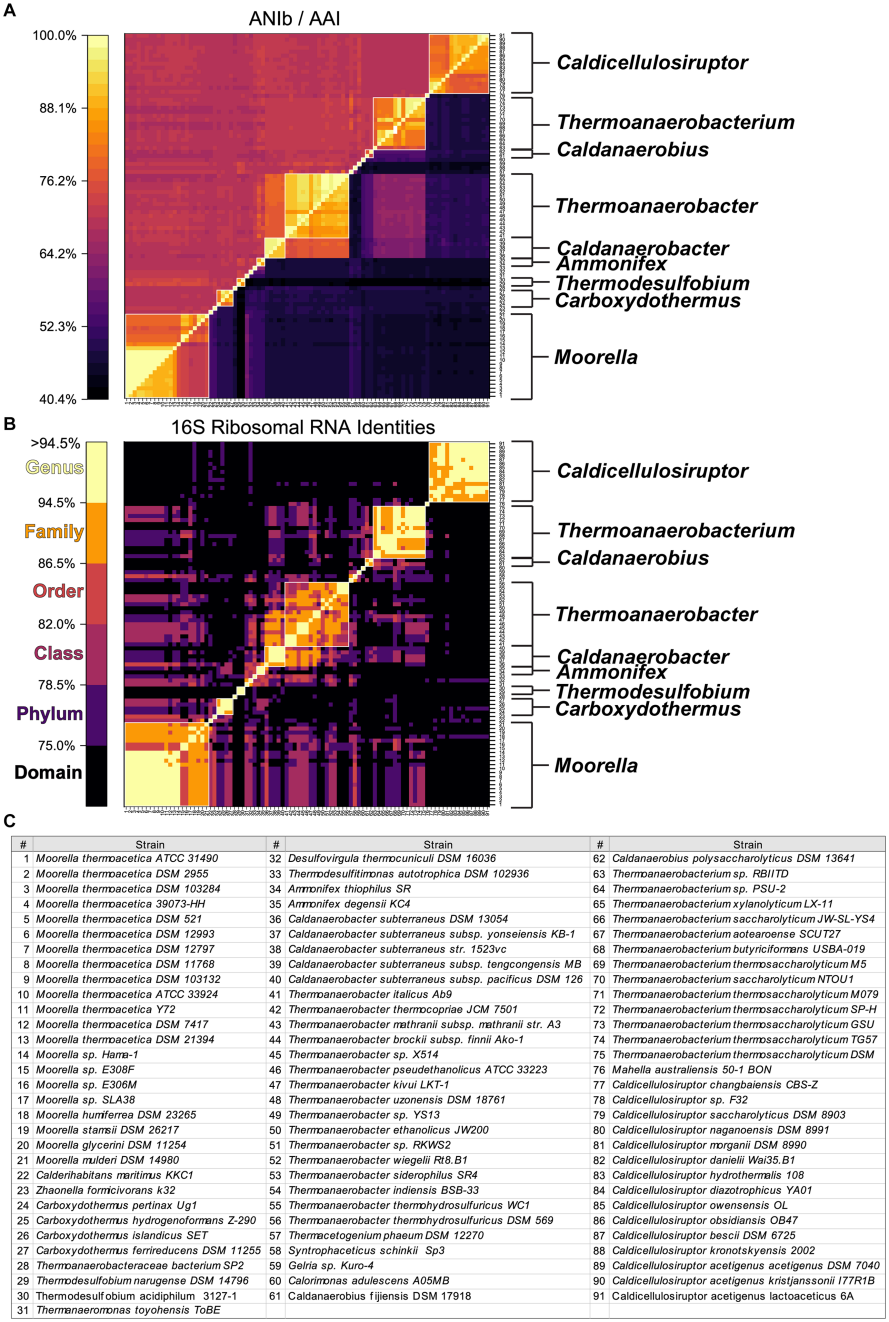
Identity matrix assessment of 91 currently classified Thermoanaerobacterales. **(A)** Whole-genome average nucleotide identity (ANIb, upper left triangle) and average amino acid identity (AAI, lower left triangle). **(B)** 16S ribosomal RNA identities from CULSTAL-Omega alignment, color map of taxonomic ranks is based on “Minimum Sequence ID” (Yarza et al., 2014). **(C)** Strain name to number key for **(A,B)**. White outline boxes indicated current NCBI genus classifications, genera with more than 1 strain present are labeled to the left of each matrix in **(A,B)**.

Reclassification at the species level is further supported by Average Nucleotide Identity (ANI) and Average Amino Acid Identity (AAI) comparisons of genome-sequenced strains currently assigned to the Order Thermoanaerobacterales (Figure 2), including 15 strains within the genus *Caldicellulosiruptor* (Table 1). Color coding in Figure 2B reflects previously proposed taxonomic grouping thresholds for 16S rRNA gene sequence identities (Yarza et al., 2014). Although the 16S rRNA gene sequence analysis is less clear than ANI/AAI, as expected, only a few current Thermoanaerobacterales genera are indicated as having Order level or lower relatedness by 16S rRNA.

**Figure 3:**
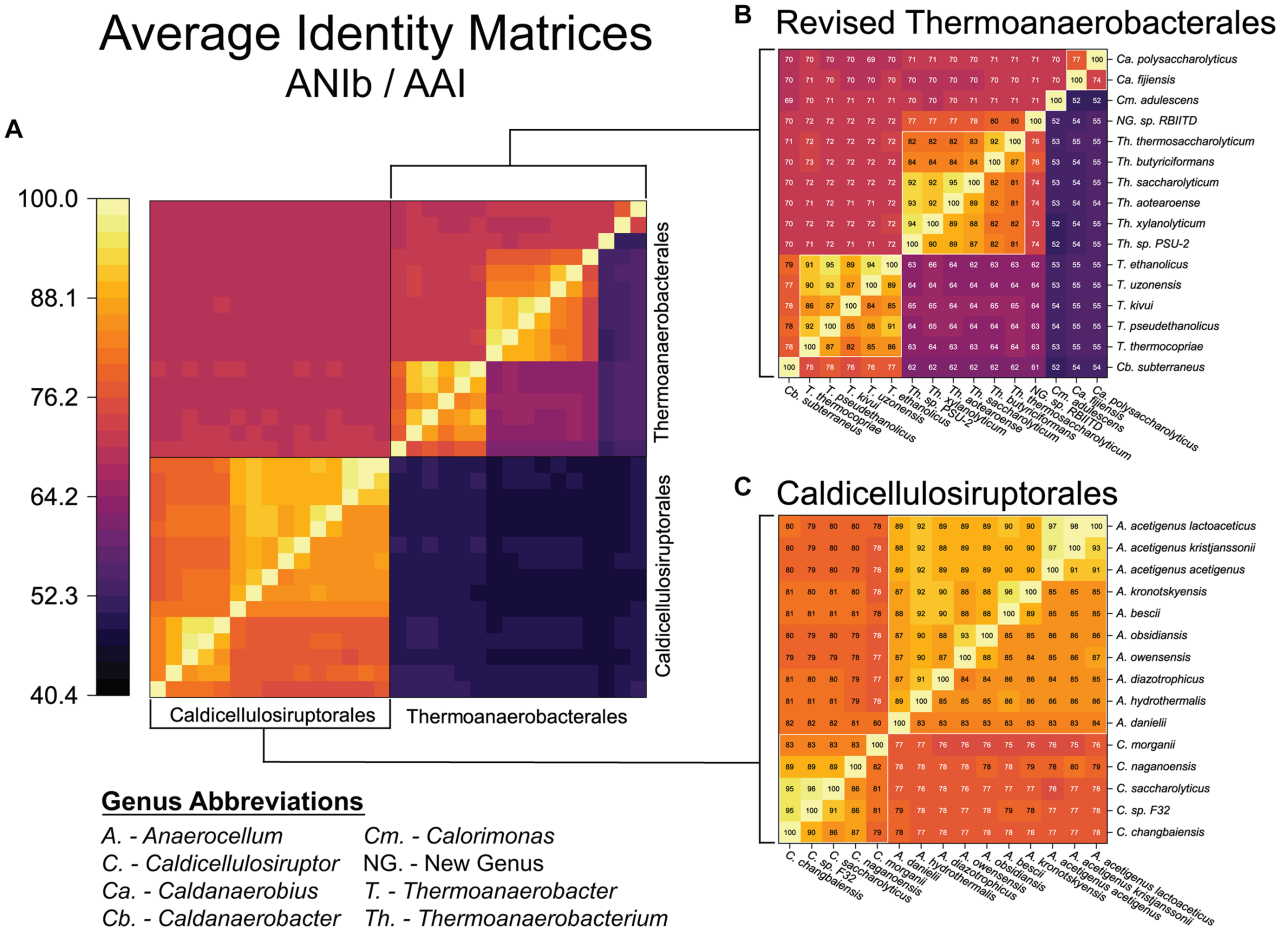
Genome-wide (ANIb/AAI) assessment of the reclassified orders: Caldicellulosiruptorales and Thermoanaerobacterales. **(A)** Whole genome average nucleotide identity (ANIb, upper left triangle) and average amino acid identity (AAI, lower right triangle) for the revised Thermoanaerobacterales and Caldicellulosiruptorales (color heatmap scale is the same used in Figure 2). Expanded views with details shown in **(B)** Thermoanaerobacterales and **(C)** Caldicellulosiruptorales. White boxes represent updated genus classifications.

To further investigate the proposed re-classification of the Thermoanaerobacterales, a genome-wide assessment was done (Figure 3). To investigate species-level classification, we assume that ANI values ≥94–96% are indicative of strains of the same species (Richter and Rossello-Mora, 2009; Kim et al., 2014). ANI/AAI results indicate several species from the genera *Thermoanaerobacter, Moorella, Carboxythermus, Thermoanaerobacterium, Caldicellulosiruptor*, and the new genus *Anaerocellum* need to be consolidated as strains of previously described species (Table 2). For example, *Thermoanaerobacter ethanolicus* (type strain JW200, the first isolate from this group) should now include an additional 6 strains currently assigned as *T. thermohydrosulfuricus* WC1, *T. thermohydrosulfuricus* DSM569, *T.* sp. RKWS2, *T. siderophilus* SR4, *T. weigelii* RT8.B1, and *T. indiensis* BSB-33, all of which share ANIb ≥97%.

**Table 2:** Proposed species-level reclassification of species currently assigned to order Thermoanaerobacterales.

Proposed species name	Strain (^T^type)	Heterotypic synonyms based on NCBI taxonomy (March 2023)	Justification/notes
*Thermoanaerobacter ethanolicus*	JW200^T^	*Thermoanaerobacter ethanolicus*	ANI/AAI (97–99/90–94%) for all strains general agreement with GTDB-tk. *T. weigelli* listed as separate species in ~5,000aa MSA GTDB taxonomy, but has >97% ANI with all strains and agrees with ~20,000aa MSA GTDB-tk analysis.
	SR4	*Thermoanaerobacter siderophilus*	
	BSB-33	*Thermoanaerobacter indiensis*	
	WC1	*Thermoanaerobacter thermohydrosulfuricus*	
	L77-66	*Thermoanaerobacter thermohydrosulfuricus*	
	RT8.B1	*Thermoanaerobacter weigelii*	
	RKWS2	*Thermoanaerobacter* sp. RKWS2	
*Thermoanaerobacter pseudethanolicus*	39E^T^	*Thermoanaerobacter pseudethanolicus*	ANI/AAI (98–100/94–96%). GTDB-tk agreement.
	AKo-1	*Thermoanaerobacter brockii subsp. finnii*	
	X514	*Thermoanaerobacter* sp. X514	
*Thermoanaerobacter uzonensis*	JW/IW-010^T^	*Thermoanaerobacter uzonensis*	ANI/AAI (97/91%). GTDB-tk agreement.
	YS13	*Thermoanaerobacter* sp. YS13	
*Thermoanaerobacter thermocopriae*	JT3-3^T^	*Thermoanaerobacter thermocopriae*	ANI/AAI (98–99/93%). GTDB-tk agreement.
	Ab9	*Thermoanaerobacter italicus*	
	A3	*Thermoanaerobacter mathranii subsp. mathranii*	
New species	AMP^T^	*Moorella thermoacetica*	GTDB-tk agreement. ANI/AAI (93/85%) support split from other *M. thermoacetica* as a new species.
*Moorella* sp. Hama-1	Hama-1^T^	*Moorella* sp. Hama-1	ANI/AAI & GTDB-tk agreement
*Moorella* sp. E308F	E308F^T^	*Moorella* sp. E308F	ANI/AAI (99/90%), GTDB-tk agreement.
	E306M	*Moorella* sp. E306M	
*Moorella* sp. SLA38	SLA38^T^	*Moorella* sp. SLA38	ANI/AAI, GTDB-tk agreement.
*Carboxydothermus hydrogenoformans*	Z-2901^T^	*Carboxydothermus hydrogenoformans*	ANI/AAI (98/91%). GTDB-tk agreement.
	JW/AS-Y7	*Carboxydothermus ferrireducens*	
*Thermoanaerobacterium aotearoense*	SCUT27^T^	*Thermoanaerobacterium aotearoense*	ANI/AAI (100/94%), GTDB-tk agreement. *T. saccharolyticum* NTOU1 and JW-SL-YS485 are separated
	JW-SL-YS485	*Thermoanaerobacterium saccharolyticum*	
*Thermoanaerobacterium* sp. PSU-2	PSU-2^T^	*Thermoanaerobacterium* sp. PSU-2	ANI/AAI and GTDB-tk agreement
New genus sp. RBIITD	RBIITD^T^	*Thermoanaerobacterium* sp. RBIITD	ANI/AAI/GTDB-tk indicated as species of a new genus.
*Biomaibacter acetigenes*	SP2	Thermoanaerobacteraceae bacterium SP2	~5,000aa MSA GTDB taxonomy placed this unclassified Thermoanaerobacterales as *B. acetigenes*
*Syntrophomonas* sp. UBA4844	Kuro-4	*Gelria* sp. Kuro-4	*Gelria* sp. Kuro-4 is likely a member of *Syntrophomonas* based on ~5,000aa MSA GTDB taxonomy.
*Caldicellulosiruptor saccharolyticus*	Tp 8 T.6.3.3.1 ^T^	*Caldicellulosiruptor saccharolyticus*	ANI/AAI: *Csac*/*C*F32 (96/91%), Csac/Ccha: (95/86%), Ccha/CF32: (95/90%) GTDB-tk agreement
	CBZ	*Caldicellulosiruptor changbaiensis*	
	F32	*Caldicellulosiruptor* sp. F32	
*Anaerocellum bescii*	Z-1320^T^	*Anaerocellum bescii*	*Abes/Akro* ANI/AAI (96/89%). GTDB-tk agreement.
	2002	*Anaerocellum kronotskyensis* 2002	

Within the Caldicellulosiruptorales, several species level reclassifications are appropriate. The recently proposed reclassifying of *A. lactoaceticus* and *A. kristjanssonii* as subspecies of *A. acetigenus* (Habib et al., 2021) is supported by ANI values all ≥97% (Figure 3). Note that *A. acetigenus* was previously reclassified from *Thermoanaerobium acetigenum* based on 16S rRNA gene sequence and physiological properties (Onyenwoke et al., 2006). What we now term *A. bescii* and *A. kronotskyensis* should be considered the same species, given that their ANI is 96% (Table 2; Figure 3); as such, *A.* kronotskyensis 2002 is designated as *A. bescii* strain 2002. Two other *kronotskyensis* strains (2006, 2,902) were classified as this specices based on 16S rRNA (Miroshnichenko et al., 2008) but have no available genome sequences. These may also be strains of *A. bescii,* but without genome sequences this cannot be verified.

Note that the inter-genus ANI of the Caldicellulosiruptorales is ≤82%, indicative of the divergence between the genera *Anaerocellum* and *Caldicellulosiruptor*, established by the GTDB-tk analysis. The *Caldicellulosiruptor* genus share an ANIb >83% (89% if *C. morganii* is excluded; Figure 3). Note that *C. morganii* is the most divergent of the Caldicellulosiruptorales by ANI/AAI, although the bac120 (GTDB-tk) phylogenetic tree places it squarely within the *Caldicellulosiruptor* genus (Figure 1). A case can be made that *C. saccharoloyticus*, *C.* sp. F32, and *C. changbaiensis* CBZ are the same species (ANI’s ≥ 95% and agreement with bac120 phylogenetic inference; Table 2); in fact, *C. saccharolyticus* and *C.* sp. F32 have been designated as such by the China General Microbiological Culture Collection (CGMCC). However, the *C.* sp. F32 genome is currently in 127 contigs, thus lowering the confidence in its taxonomic placement compared to other *Caldicellulosiruptor* strains.

The core genomes of various taxonomic levels were evaluated in the process of whole-genome analysis. The core and pan genomes of the Thermoanaerobacteraceae are 306 / 6,915 for the family, 1,308 / 3,510 for *Caldanaerobacter*, 815 / 4,087 for *Thermoanaerobacter*, and 1,374 / 4,419 for *Thermoanaerobacterium*. Within the Caldicellulosiruptoraceae, the core and pan genomes are: 1,248 / 3,833 for the family, 1,496 / 3,527 for *Anaerocellum*, and 1,367 / 3,027 for *Caldicellulosiruptor* (Supplementary Figure S1). The pangenome of the Caldicellulosiruptoraceae remains open, implying more genetic diversity within the Family remains to be discovered.

The core and pan genomes of the Thermoanaerobacteraceae, respectively are: 306/6,915 for the family, 1,308/3,510 for *Caldanaerobacter,* 815/4,087 for *Thermoanaerobacter,* and 1,374/4,419 for *Thermoanaerobacterium* (Supplementary Figure S1).

### 3.2. Global distribution of the order Caldicellulosiruptorales and relationship to taxonomy

**Figure 4:**
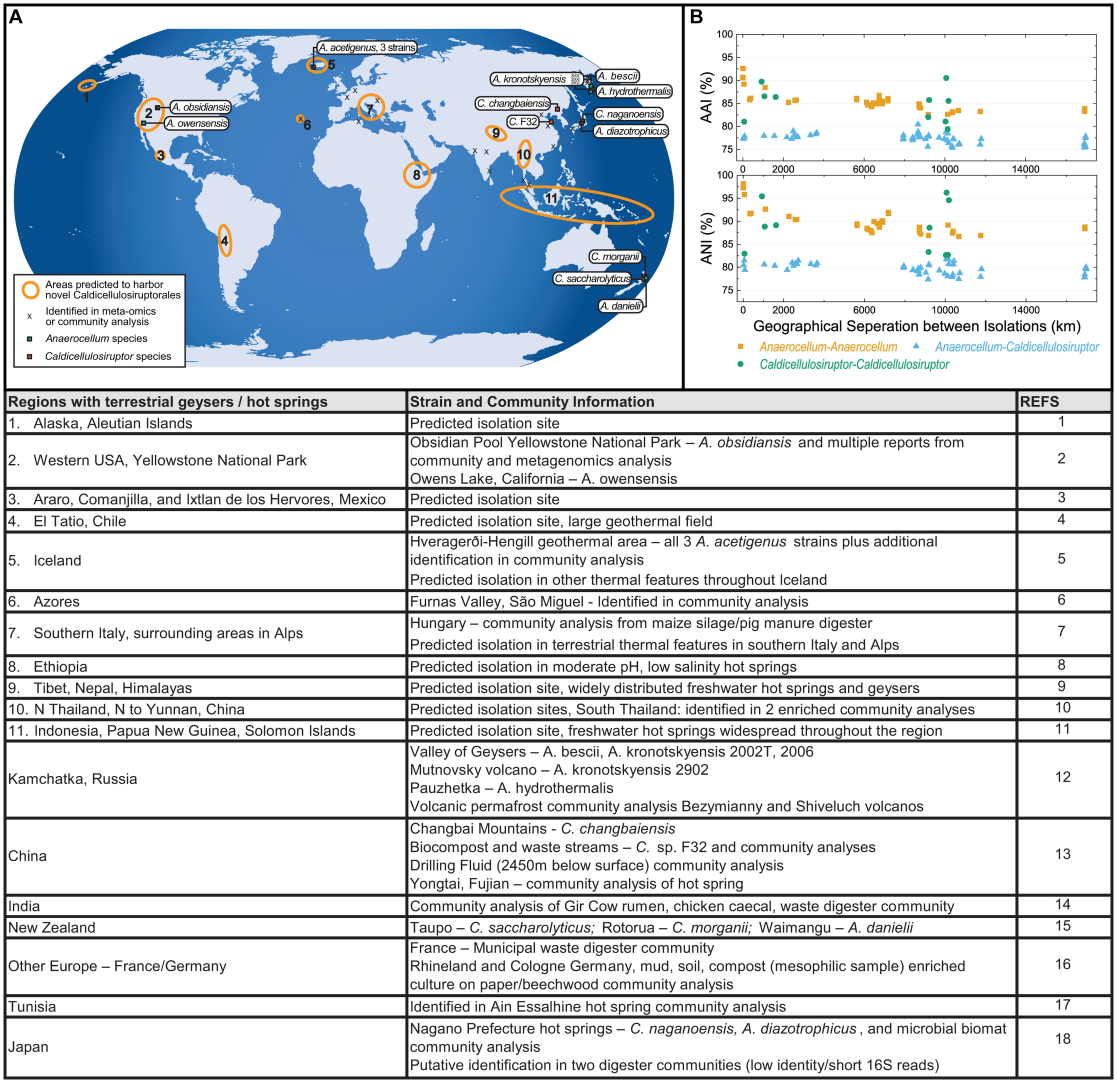
Biogeographic distribution of current and putative isolation sites for Caldicellulosiruptorales. **(A)** Strains with genome sequences are labeled, and isolation sites marked. Presence of Caldicellulosiruptorales in multi-omics and community analysis are marked with “X.” Circled areas are predicted to harbor Caldicellulosiruptorales. Listed in the table below are details of these areas and isolation sites. **(B)** Whole genome average amino acid identity (AAI) and average nucleotide identity (ANI) for strain pairs with genome sequences plotted against the distance separating their isolation sites; color coded based on pairings within and outside the two proposed genera (*Caldicellulosiruptor* and *Anaerocellum*). Reference list: 1 - (Alaska Department of Natural Resources, 1984); 2 - (Berry et al., 1980; Elkins et al., 2010; Hamilton-Brehm et al., 2010; Vishnivetskaya et al., 2015; Lee et al., 2018); 3, 8, 9, 11 - (Johnson, 2010); 4 - (Fernandez-Turiel et al., 2005); 5 - (Nielsen, 1993; Mladenovska et al., 1995; Sonne-Hansen and Ahring, 1997; Bredholt et al., 1999; Onyenwoke et al., 2006; Orlygsson et al., 2010); 6 - (Sahm et al., 2013); 7 - (Johnson, 2010; Wirth et al., 2012); 10 - (Johnson, 2010; Hniman et al., 2011); 12- (Rainey et al., 1993; Miroshnichenko et al., 2008; Sahm et al., 2013; Vishnivetskaya et al., 2015, 2022); 13 - (Zhang et al., 2007, 2016; Qiu et al., 2011; Ying et al., 2013; Bing et al., 2015; Liu et al., 2015); 14 - (Pandit et al., 2016, 2018a,b); 15 - (Sissons et al., 1987; Rainey et al., 1993, 1994; Lee et al., 2015, 2018); 16 - (Svetlitchnyi et al., 2013; Lu et al., 2014); 17 - (Sayeh et al., 2010); 18 - (Taya et al., 1988; Rainey et al., 1993; Narihiro et al., 2009; Lee et al., 2015, 2018; Cheng et al., 2018; Nishihara et al., 2018; Chen et al., 2021a,b).

The wide global distribution of the Caldicellulosiruptorales is evident from the isolation sites of currently named species as well as from signatures detected in community analyses (Figure 4; Blumer-Schuette, 2020). Given the closely related microbiological features of members of the Caldicellulosiruptorales, it is interesting to consider how these species became globally distributed and the relationships between geography, physiochemical features of isolation sites, and strain relatedness. The fact that all known members of the Caldicellulosiruptorales grow best at temperatures above 70°C differentiates them from almost all other characterized bacteria, which are mostly mesophilic or moderately thermophilic (T_opt_ ≤ 65°C). Community analyses of terrestrial hot springs indicate that, above 65°C, Caldicellulosiruptorales dominate (Vishnivetskaya et al., 2015; Lee et al., 2018). Based on the global presence of Caldicellulosiruptorales in low-salinity terrestrial thermal sites (>65°C) and the relatedness of strains isolated in various regions, additional areas likely to harbor novel strains of Caldicellulosiruptorales can be identified. These areas are detailed in Figure 4, both circled in the map (Figure 4A) with details provided below the map. There is a notable absence of isolated Caldicellulosiruptorales from Africa and South America, although there are thermal features on these continents hospitable to Caldicellulosiruptorales. Presence of widespread thermal features and high similarity of strains currently isolated from North America and Iceland may indicate that additional more divergent Caldicellulosiruptorales exist in these areas, which would reflect the diversity seen in China, Kamchatka, Japan, and New Zealand.

It is also interesting to consider how the globally distributed Caldicellulosiruptorales compare with respect to geographic separation of isolation sites and overall genetic relatedness. Figure 4B shows how geographical distance between isolation sites for Caldicellulosiruptorales (Supplementary Table S2) relates to AAI and ANI. While it is not surprising that species and strains isolated from immediate proximity to each other can have high AAI/ANI values, it is interesting that some species from isolation sites separated by thousands of kilometers are also closely related. Further, several strains isolated from relatively close sites have lower AAI/ANI values, indicating close proximity of isolation does not always infer high strain similarity even within the same genus. Thus, from available genomic data, there is no correlation between geographical spacing of isolation sites and ANI/AAI for the Caldicellulosiruptorales, at least at for distance >50 km. Presumably, at some smaller distance (i.e., <50 km), there could be a correlation, where multiple strains from a single geothermal feature, or very near-by thermal features, could have high relatedness. The Icelandic strains (*A. acetigenus*) may hint at this possibility due to their high relatedness and close proximity of isolation. However, at the same time, species isolated <100 km from each other have much lower relatedness, like those from New Zealand (*C. morganii, C. saccharolyticus, A. danielii*) or Japan (*C. naganoensis, A. diazotrophicus*).

### 3.3. Carbohydrate utilization in the Caldicellulosiruptorales and Thermoanaerobacterales

**Figure 5:**
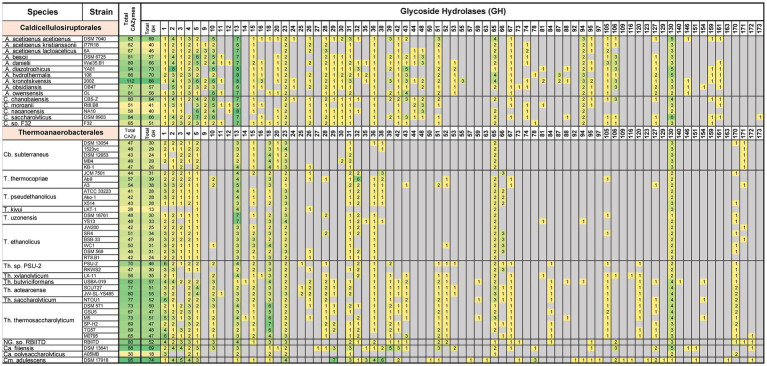
Distribution of glycoside hydrolases among the Caldicellulosiruptorales and Thermoanaerobacterales. Boxes indicate number of coding sequences (CDS) that contain at least one of the indicated GH domain family. CDS were only counted once, even if the CDS contained multiple domains. Color coding heat map was applied based on the number of CDS present for the respective enzyme classes. Gray boxes indicate that class of GH was not detected (value of 0). Genus-species nomenclature reflects taxonomy changes proposed here. *A: Anaerocellum, C: Caldicellulosiruptor, Cb: Caldanaerobius, T: Thermoanaerobacter, Th: Thermoanaerobacterium, Ca: Caldanaerobacter, Cm: Calorimonas,* NG: new genus.

Comparing and contrasting the underlying metabolism, physiology, and ecology of microorganisms goes beyond 16S rRNA, GTDB-tk, and ANI/AAI analyses. Interest in the Caldicellulosiruptorales was initially driven by their ability to degrade lignocellulosic biomass and the inventory of carbohydrate active enzymes (CAZymes) supporting this characteristic (Blumer-Schuette et al., 2014). Specifically, within the group of CAZymes are intracellular, extracellular, and surface-layer Glycoside Hydrolases (GHs) that process carbohydrates; many of these enzymes have multiple domains and associated carbohydrate binding modules (CBMs; Blumer-Schuette et al., 2010; Conway et al., 2016, 2017, 2018; Crosby et al., 2022; Laemthong et al., 2022). Figure 5 summarizes GH inventories separated by GH family (Drula et al., 2022), for the 15 genome-sequenced Caldicellulosiruptorales and 37 sequenced strains within the revised Thermoanaerobacterales. It is evident that the GH inventory varies widely across genera and species, and even across strains within a species. To this point, *A. acetigenus* str*. Acetigenus* has 15 or more total CAZymes, and 20 or more GH containing CAZymes, than either of the other two strains of this species.

A wide variety of GH family domains are found in all Caldicellulosiruptorales genomes; collectively these enable degradation of carbohydrates found in lignocellulosic biomass to recruit them as carbon and energy sources. Enzyme functions likely associated with these domains include xylan hydrolysis, exo- and endo-acting cleavage of β-glycans, hydrolysis of α-glucans (such as starch, pullulan), pectin degradation, hydrolysis of galactomannans, mono-, di-, and oligosaccharide phosphorylation, and hydrolysis of peptidoglycan or chitin. Domains related to cleavage of terminal glucose residues from β-glycosides (GH1) and α-glucans (GH15), as well as GH51 (likely endo acting β-glucanase or α-L-arabinofuranosidase), are found in all but one species. Domains related to phosphorylases (GH65), uncapping of glucuronic acid decorated xylooligosaccharides (GH67), and hydrolysis of unsaturated glucuronyl/galacturonyl linkages (GH105), and GH4 (possible α-glucosidase, galactosidase, glucuronidase, or galacturonase) are present in all but two species. GH9 and GH48 domains (and CBM3) are central to enzymes that catalyze microcrystalline cellulose hydrolysis, notably present in the multi-domain cellulase, CelA (Conway et al., 2017) one or both of these GH domains are present in all five *Caldicellulosiruptor* species and six of ten *Anaerocellum* species. Strains lacking these cellulases are unable to grow on microcrystalline cellulose (Avicel). Of note here, *A. diazotrophicus* (which lacks these enzymes) was reported in its isolation paper to grow on Avicel (Chen et al., 2021b). However, as part of this work, *A. diazotrophicus* was readily cultured on cellobiose, beechwood xylan, and corn fiber, but no growth was observed on Avicel at 75°C. This result reinforces that Caldicellulosiruptorales lacking GH9 and GH48 domains cannot grow on microcrystalline cellulose. Both strongly cellulolytic and non-cellulolytic (or weakly cellulolytic) species are found in most Caldicellulosiruptorales isolation biotopes (Vishnivetskaya et al., 2015; Lee et al., 2018). This reflects the cooperative nature of microbial communities. For example, not all species in a biotope need to be cellulolytic to utilize lignocellulose, but instead some species can degrade cellulose through extracellular GHs thereby enabling scavenging of the resulting oligosaccharides by non-cellulolytic species. Interestingly, common to all members of the Caldicellulosiruptorales are peptidoglycan lyases / chitinase (GH23) and peptidoglycan/ chitin binding domains (CBM50). This implicates degradation of bacterial or fungal cell wall remnants to support acquisition of substrates in otherwise nutritionally sparse biotopes.

In the Thermoanaerobacterales genomes, highly represented GH families (GH1-5, GH13, GH15, GH23, GH31, GH36, GH65, GH130) mirror what is seen in the Caldicellulosiruptorales, with two exceptions. GH18 domains (chitinases, lysozymes) are common to the Thermoanaerobacterales, but absent in the Caldicellulosiruptorales. None of the genome sequenced Thermoanaerobacterales are known to degrade crystalline cellulose, and accordingly, none contain genes encoding GH9, GH48, and CBM3 domains, which are important for microcrystalline cellulose degradation. It is also interesting that many Thermoanaerobacterales lack GH10/11 domains that relate to xylan hydrolysis (*Thermoanaerobacterium, Caldanaerobius polysaccharolyticus,* and some *Thermoanaerobacter* are exceptions). Among the Thermoanaerobacterales, *Thermoanaerobacterium* have the most diversity in CAZymes, reflecting their hemicellulolytic activity. Similar trends are seen with other CAZyme domains (carbohydrate binding modules, polysaccharide lysases, and carbohydrate esterases; Supplementary Figure S2). Based on CAZyme inventory, Caldicellulosiruptorales have a broader carbohydrate appetite than the Thermoanaerobacterales, especially with respect to crystalline cellulose.

## 4. Discussion

Clearly, keeping taxonomy up to date in the face of expanding genome sequence databases is challenging. Fortunately, bioinformatic analyses, using tools such as GTDB-tk, ANI, and AAI, can be used to update taxonomy to more accurately reflect phylogeny, although heuristics and criteria for classification have not been universally established. Many fermentative anaerobes were isolated decades ago, prior to the use of whole genome sequence data, such that their taxonomy required updating. While classifications with 16S rRNA sequencing greatly improved taxonomy beyond phenotypic classifications, many misclassifications persist. The Genome Taxonomy Database “de_novo_wf” workflow, using 120 markers for bacteria and ~ 24,000 amino acid alignment, allows for classifications above the species genus level to be readily discerned. This work shows, at least for the former Thermoanaerobacterales, that at the species level and below, whole-genome comparison (ANI/AAI) is needed for accurate reflection of phylogeny by taxonomy. GTDB has reasonable accuracy at and below the genus level, but several disagreements between the available GTDB taxonomy (~5,000 amino acid alignment) and the expanded GTDB-tk (~24,000 amino acid alignment, “de_novo_wf”) and ANI/AAI were found. These include the split of the genus *Caldicellulosiruptor* and species/strain designations of *T. weigelii* and *A. kronotskyensis*. A robust method for taxonomic classification should go beyond 16S rRNA identities and published GTDB taxonomy by using GTDB-tk for accurate classification down the species level, and then use ANI/AAI to confirm and reinforce species/strain placement.

Efforts to update the taxonomy of NCBI classified Thermoanaerobacterales to better reflect their phylogeny shows that many strains do not belong in the Order Thermoanaerobacterales. Among the findings are that the Caldicellulosiruptorales occupy a distinct phylogenetic niche that differentiates them from the Thermoanaerobacterales. Furthermore, the genus *Caldicellulosiruptor* should be divided into two genera: *Caldicellulosiruptor* and *Anaerocellum*. Reflected in the distance between these Orders is their thermophilicity; Caldicellulosiruptorales preference for higher temperature allows them to occupy biotopes distinct from the Thermoanaerobacterales. Previous examination of a wide range of lignocellulosic plant biomasses revealed that Thermoanaerobacterales were present but dormant at ambient temperatures, and could be stimulated and grown up to about 65°C. However, no evidence of Caldicellulosiruptorales were indicated in any of the biomasses (Bing et al., 2023a), and a threshold for growth of indigenous microbial life harbored within plant biomass (≤ 70°C) was proposed.

The Caldicellulosiruptorales are asporogenous, such that their dispersal would not involve transport of spores. However, they are dormant at lower temperatures and, like other asporogenous extreme thermophiles, can remain in a viable state even after exposure to low temperatures for long times (Milojevic et al., 2022). The dispersal of ‘thermospores’ through ocean currents may explain the wide distribution of moderate thermophiles in marine environments world-wide. Moreover, asporogenous extreme thermophiles associated with ocean floor black smokers and other types of thermal vents could disperse through oceanic fluid motion and microbial motility (Milojevic et al., 2022). However, how terrestrial extreme thermophiles, such as the Caldicellulosiruptorales, spread globally is less clear. Certainly, volcanic activity and aerosols thereby formed could serve as a vector for dispersal through clouds and atmospheric processes (Šantl-Temkiv et al., 2022; Vishnivetskaya et al., 2022). This is likely the best explanation for the global distribution of Caldicellulosiruptorales and may explain how metagenomic signatures of these bacteria have been found in volcanic ash permafrost and non-thermal, terrestrial environments (Figure 4). As is evident from Figure 4, many thermal, pH-neutral terrestrial biotopes that could harbor Caldicellulosiruptorales are yet unexplored and metagenomic analysis of these sites could prove useful in validating the proposed revisions to taxonomy; exploration of surrounding non-thermal features could gain further insight into the dispersal of terrestrial extreme thermophiles.

Ultimately, small segments of the genome may define microbiological characteristics that cannot be gleaned from global genomic analysis (such as carbohydrate metabolism, discussed in part here through analysis of CAZymes, but extends to other genes with high phenotypic influence, such as toxin-antitoxin biofilm regulation (Lewis et al., 2023)). We show here that when carbohydrate “appetite” is mapped to CAZyme inventory, the Caldicellulosiruptorales differ from the Thermoanaerobacterales in that many species of the former, but not the latter, can degrade microcrystalline cellulose. Most geographical regions harbor both cellulolytic and weak/non-cellulolytic Caldicellulosiruptorales, which could suggest a possible existence of Caldicellulosiruptorales communities, where the non-cellulolytic species benefit from cellulases secreted by the cellulolytic species. In extremely thermophilic terrestrial environments, these non-cellulolytic Caldicellulosiruptorales potentially fill the environmental niche occupied at lower temperatures by other hemicellulolytic and oligosaccharide-consuming organisms, such as *Thermoanaerobacterium* and *Thermoanaerobacter* species. The latter two genera are often found along with cellulolytic Clostridia (such as *Thermoclostridium stercorarium* and *Acetivibrio thermocellus*), forming moderately thermophilic plant biomass degrading communities with diversity spanning multiple Orders (Liu et al., 2008; Bing et al., 2023a). Further, metagenomic analyses to date indicate that microbial biodiversity in terrestrial hot springs decreases with increasing temperature (Lee et al., 2018), such that the gene pool becomes more limited. In line with this, Caldicellulosiruptorales seem to be the only plant biomass degraders in their higher temperature (≥70°C) environments (based on available isolation and metagenomics reports for these thermal features). Caldicellulosiruptorales seem to have evolved to occupy multiple niches normally filled by multiple Orders of bacteria at lower temperatures. Due to the more limited microbial diversity in these extreme temperatures, Caldicellulosiruptorales species have evolved to harbor a broad array of CAZymes, allowing them to degrade and consume a wide range of polysaccharides, as the presence of other polysaccharide degrading microorganisms is less likely.

Specialization of Caldicellulosiruptorales to their localized environment might drive the gain and loss of CAZymes. How the Caldicellulosiruptorales acquire new CAZymes is not at all understood. The majority of their CAZymes have low homology (*via* NCBI BLAST search) outside of the Caldicellulosiruptorales, and homologous CAZymes within Caldicellulosiruptorales have similar average percent amino acid identities to that of the whole-genome AAI (Supplementary Figure S3), suggesting vertical gene transfer and evolution is significant for these CAZymes. Caldicellulosiruptoraceae all contain a plethora of transposases encoded in their genomes; this could also potentially aid in the movement of genes, including CAZymes, within their own genomes or between other Caldicellulosiruptorales. Possible evidence for this is found in species with fragmented glucan degradation loci (GDL), including *A. acetigenus* (all three strains) and *C. morganii* (Lee et al., 2018), where transposases are annotated immediately adjacent to GDL fragments. A more detailed evaluation of the role of transposases in the Caldicellulosiruptorales and their communities is obviously needed.

Caldicellulosiruptorales clearly have evolved to fit their environmental niche, where they excel at scavenging a variety of carbohydrates available in their environment. Indeed, CAZyme inventory of the Caldicellulosiruptorales reflect the types of biomasses common to their locales including (hemi)celluloses, pectin, and starches found in woods and grasses, as well as their fruits and seeds. Less obvious sources of carbohydrate could include chitin or peptidoglycan in fungal/bacterial cell walls, or lichenin (β1,3; β1,4 glucan) and galactomannan rich organisms like mosses and lichens, including Icelandic moss [which occurs outside of Iceland in most regions containing Caldicellulosiruptorales, (Table 1; Ingólfsdóttir, 2000)]. CAZyme inventory of Caldicellulosiruptorales varies by species; some like *A. kronotskyensis* have broad appetites for available carbohydrates. *A. kronotskyensis* contains 44 out of the 51 GH domains present in the Caldicellulosiruptorales pangenome and those missing GH domains are relatively rare across the family. Other Caldicellulosiruptorales that lack cellulases, or have fewer CAZymes, either live in environments with more limited sources of carbohydrates, or have evolved to live in communities with other strains, occupying narrower ecological roles. The latter can be seen for *A. acetigenus* strains, which vary widely in CAZyme inventory despite highly localized geographic proximity and high genome-wide identity. It is possible that these strains may exist in communities together where only a subpopulation degrade cellulose (such as *A. acetigenus lactoaceticus*). However, in order to prove this, a more detailed evaluation of Caldicellulosiruptorales communities is needed. In context of major research interests in the Caldicellulosiruptorales related to lignocellulose degradation, novel CAZymes likely still exist in environments not yet sampled, harbored in undiscovered strains. Consideration of polysaccharides local to isolation environments could aid in finding CAZymes or strains capable of degrading specific substrates.

Beyond CAZymes, the most differentiating feature between the Caldicellulosiruptorales and Thermoanaerobacterales is thermophilicity, given that the differences in optimal growth temperatures exceed 10°C. This is reflected by the AAI of homologous proteins, where increased protein thermostability likely maps to changes in amino acid sequence to maintain function at higher temperatures. As many aspects of fermentative anaerobic metabolism should be highly conserved, many proteins must evolve to become more (or less) thermostable. As such, the revised taxonomy containing two Orders (Caldicellulosiruptorales and Thermoanaerobacterales) reflects this phylogenic divergence. While the revisions here improve the taxonomy of the Thermoanaerobacterales and Caldicellulosiruptorales, many unresolved taxonomic issues still remain for strains no longer a part of the Thermoanaerobacterales, as well as Class and Phyla classifications for the Clostridia. In any event, bioinformatic tools are useful for taxonomy and phylogeny, but phenotype and ecology must still lean heavily on microbiological details.

## Data availability statement

The datasets presented in this study can be found in online repositories. The names of the repository/repositories and accession number(s) can be found in the article/Supplementary material.

## Author contributions

RB and RK conceived the study. RB, DW, JC, MA, and RK carried out the experiments and analysis. RB, MA, and RK wrote the manuscript. All authors contributed to the article and approved the submitted version.

## Funding

This work was supported by the US Department of Energy BER Awards DE-SC0019391 and DE-SC0022192. JC acknowledges support from a US DoEd GAANN Fellowship (P200A160061). RB and DW acknowledge support from an NIH Biotechnology Traineeship (NIH T32 GM008776-16).

## Conflict of interest

The authors declare that the research was conducted in the absence of any commercial or financial relationships that could be construed as a potential conflict of interest.

## Publisher’s note

All claims expressed in this article are solely those of the authors and do not necessarily represent those of their affiliated organizations, or those of the publisher, the editors and the reviewers. Any product that may be evaluated in this article, or claim that may be made by its manufacturer, is not guaranteed or endorsed by the publisher.

## Footnotes

^1^https://stevemorse.org/nearest/distancebatch.html

^2^https://vis4.net/palettes
