# Supplementary material for: Whither the genus Caldicellulosiruptor and the order Thermoanaerobacterales: phylogeny, taxonomy, ecology, and phenotype
Source: Front Microbiol. 2023 Aug 3;14:1212538. doi: 10.3389/fmicb.2023.1212538 (PMC10434631; doi:10.3389/fmicb.2023.1212538)
Supplement: Supplementary file 3 [file Image_1.PDF]

# Figure S1. Core, Soft Core, and Pan-Genomes

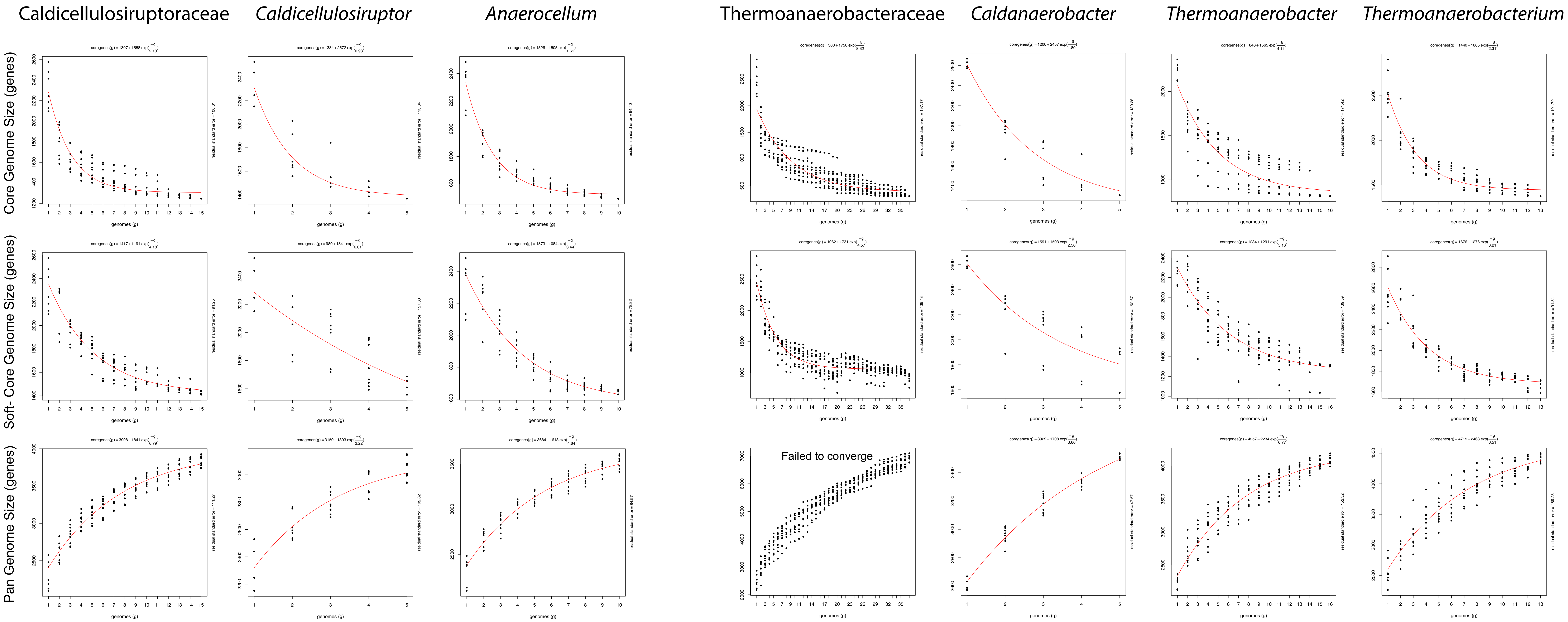


| | Genomes | Core | Soft-Core | Pan |
|---|---|---|---|---|
| **Caldicellulosiruptoraceae** | 15 | 1248 | 1437±12 | 3833±67 |
| ***Caldicellulosiruptor*** | 5 | 1367 | 1633±41 | 3027±80 |
| ***Anaerocellum*** | 10 | 1496 | 1647±13 | 3527±63 |

| | Genomes | Core | Soft-Core | Pan |
|---|---|---|---|---|
| **Thermoanaerobacteraceae** | 37 | 306 | 921±70 | 6915±118 |
| ***Caldanaerobacter*** | 5 | 1308 | 1769±167 | 3510±19 |
| ***Thermoanaerobacter*** | 16 | 815 | 1311±4 | 4087±63 |
| ***Thermoanaerobacterium*** | 13 | 1374 | 1687±41 | 4419±63 |
