# Supplementary material for: Whither the genus Caldicellulosiruptor and the order Thermoanaerobacterales: phylogeny, taxonomy, ecology, and phenotype
Source: Front Microbiol. 2023 Aug 3;14:1212538. doi: 10.3389/fmicb.2023.1212538 (PMC10434631; doi:10.3389/fmicb.2023.1212538)
Supplement: Supplementary file 4 [file Image_2.PDF]

| Species | Strain | Polysaccharide Lyase (PL) | | | | | | | | Carbohydrate Esterase (CE) | | | | | | | | | | Carbohydrate Binding Module (CBM) | | | | | | | | | | | | | | | | | | | | | | | | | | | | | | |
|---|---|---|---|---|---|---|---|---|---|---|---|---|---|---|---|---|---|---|---|---|---|---|---|---|---|---|---|---|---|---|---|---|---|---|---|---|---|---|---|---|---|---|---|---|---|---|---|---|---|---|
| **Caldicellulosiruptorales** | | 1 | 3 | 9 | 11 | 15 | 26 | 33 | 39 | 1 | 4 | 7 | 8 | 9 | 12 | 14 | 15 | 19 | 20 | 2 | 3 | 4 | 5 | 6 | 9 | 12 | 13 | 16 | 17 | 20 | 22 | 23 | 25 | 27 | 28 | 32 | 34 | 35 | 36 | 38 | 41 | 48 | 50 | 51 | 54 | 59 | 61 | 66 | 67 | 91 |
| A. acetigenus acetigenus | DSM 7040 |  |  |  |  |  |  |  |  |  | 5 |  | 1 | 1 |  |  |  |  | 1 |  |  | 1 |  | 2 |  |  |  |  |  | 1 | 3 |  |  |  | 1 |  |  | 1 |  |  | 1 | 3 | 3 |  |  |  |  |  |  |  |
| A. acetigenus kristjanssonii | I77R18 |  | 1 | 1 | 1 |  |  |  |  |  | 4 | 1 |  | 1 | 1 |  | 1 |  | 1 |  | 4 |  |  |  | 1 |  |  |  |  | 2 | 2 |  |  | 1 | 1 |  |  | 1 |  |  | 1 | 2 | 3 |  | 2 |  |  | 2 |  |  |
| A. acetigenus lactoaceticus | 6A |  | 1 | 1 | 2 |  |  |  |  |  | 3 |  |  | 1 | 2 |  |  |  | 1 | 2 | 6 |  |  |  | 2 |  |  |  | 1 | 3 | 2 |  |  | 2 | 1 |  |  | 2 |  |  | 2 | 3 | 4 | 1 | 2 |  |  | 2 |  |  |
| A. bescii | DSM 6725 |  | 1 | 1 | 2 |  | 1 |  |  | 1 | 4 | 1 |  | 1 | 2 |  |  |  | 1 |  | 7 |  |  | 2 |  |  |  |  |  | 2 | 4 |  |  |  | 1 |  |  | 1 | 1 |  | 1 | 2 | 3 |  | 1 |  | 1 | 2 |  |  |
| A. danielii | Wai35.B1 |  | 1 | 1 | 2 |  | 1 |  |  |  | 3 |  | 1 | 1 | 1 |  |  |  | 1 |  | 7 | 2 |  |  | 2 |  |  |  |  | 2 | 3 |  |  | 1 | 3 | 1 |  | 1 |  |  | 1 | 1 | 4 |  | 2 |  | 1 | 2 |  | 1 |
| A. diazotrophicus | YA01 | 1 |  |  | 3 |  | 1 |  |  | 1 | 4 |  | 2 | 1 | 1 |  |  |  | 1 |  |  | 2 |  | 1 | 2 |  |  |  |  | 2 | 4 |  |  |  | 2 | 2 |  | 1 |  | 2 | 1 | 1 | 4 |  | 3 |  | 2 |  |  | 1 |
| A. hydrothermalis | 108 |  |  |  | 1 |  |  |  |  |  | 4 | 1 | 1 | 1 | 1 |  |  |  | 1 |  |  | 2 |  |  | 1 |  |  |  |  | 1 | 1 |  |  | 1 | 2 | 1 |  | 4 |  |  | 1 | 1 | 4 |  | 3 |  | 2 |  | 1 |  |
| A. kronotskyensis | 2002 | 1 | 1 |  | 2 |  | 1 |  |  | 1 | 4 | 1 | 2 | 1 | 2 |  |  |  | 1 |  | 9 | 2 |  | 1 | 3 |  |  |  |  | 1 | 5 |  |  |  | 2 | 8 |  | 2 | 1 |  | 1 | 2 | 5 |  | 4 |  | 2 | 1 |  | 1 |
| A. obsidiansis | OB47 |  | 1 |  | 1 |  | 1 |  |  | 1 | 4 |  |  | 1 | 2 |  |  |  | 1 |  | 4 | 2 |  |  |  |  | 1 |  |  | 2 | 1 |  |  | 2 |  | 1 |  | 2 |  |  | 1 | 1 | 3 |  | 2 |  | 2 | 1 |  | 1 |
| A. owensensis | OL | 1 |  | 1 | 1 |  | 1 |  |  | 1 | 4 |  | 2 | 1 | 2 |  |  |  | 1 |  |  |  |  | 1 | 1 |  | 1 |  |  | 2 | 4 |  |  |  |  | 1 |  | 1 | 1 |  | 1 | 3 | 3 |  | 1 |  | 1 |  |  |  |
| C. changbaiensis | CBS-Z |  |  |  |  |  | 1 |  |  | 1 | 4 |  |  | 1 | 1 |  |  |  | 1 |  | 4 |  |  | 1 |  |  |  |  |  | 1 | 3 |  |  |  | 1 |  |  | 1 |  |  | 1 | 1 | 4 |  | 1 |  |  |  |  | 1 |
| C. morganii | Rt8.B8 |  |  | 1 | 1 |  |  |  |  |  | 2 |  |  | 1 |  | 1 |  |  | 1 |  | 7 | 2 |  |  | 2 |  | 1 |  |  | 1 | 2 |  |  |  |  | 1 |  |  | 1 |  | 1 | 1 | 2 |  | 2 |  |  |  |  |  |
| C. naganoensis | NA10 |  | 1 | 1 |  |  |  |  |  |  | 5 |  |  | 2 |  | 1 |  |  |  |  | 8 |  |  |  | 2 |  |  |  |  | 3 | 3 |  |  |  | 1 |  |  |  | 1 |  | 1 | 1 | 3 |  | 1 |  |  | 1 |  |  |
| C. saccharolyticus | DSM 8903 |  |  |  |  |  |  |  | 1 | 1 | 4 | 1 |  | 1 |  |  |  |  | 1 |  | 5 | 2 |  | 1 |  |  |  |  |  | 1 | 3 |  |  |  | 1 | 1 |  | 1 |  |  | 1 | 1 | 5 |  | 1 |  |  |  |  | 1 |
| C. sp. F32 | F32 |  |  |  |  |  |  |  |  |  | 4 | 1 |  | 1 |  | 1 |  |  | 1 |  | 2 | 2 |  |  |  |  |  |  |  | 1 | 1 |  |  |  | 1 |  |  |  | 1 |  | 1 | 1 | 4 |  | 1 |  |  |  | 1 | 1 |
| **Thermoanaerobacterales** | | 1 | 3 | 9 | 11 | 15 | 26 | 33 | 39 | 1 | 4 | 7 | 8 | 9 | 12 | 14 | 15 | 19 | 20 | 2 | 3 | 4 | 5 | 6 | 9 | 12 | 13 | 16 | 17 | 20 | 22 | 23 | 25 | 27 | 28 | 32 | 34 | 35 | 36 | 38 | 41 | 48 | 50 | 51 | 54 | 59 | 61 | 66 | 67 | 91 |
| Cb. subterraneus | DSM 13054 |  |  |  |  |  |  |  |  |  | 3 |  |  | 1 |  | 2 |  | 1 |  |  |  |  |  |  |  |  |  |  |  | 1 |  |  |  |  |  | 1 | 2 |  |  |  |  |  | 9 |  |  |  |  |  |  |  |
| | 1523vc |  |  |  |  |  |  |  |  |  | 3 |  |  | 1 |  |  |  |  |  |  |  |  |  |  |  |  |  |  |  | 1 |  |  |  |  |  |  | 1 |  |  |  |  |  | 11 |  |  |  |  |  |  |  |
| | DSM 12653 |  |  |  |  | 1 |  |  |  |  | 3 |  |  | 1 |  | 4 |  | 1 |  |  |  |  |  |  |  |  |  |  |  |  |  |  |  |  |  | 1 | 1 |  |  |  |  |  | 8 |  |  |  |  |  |  |  |
| | MB4 |  |  |  |  |  |  |  |  |  | 4 |  |  | 1 |  | 4 |  | 1 |  |  |  |  |  |  |  |  |  |  |  |  |  |  |  |  |  |  | 1 |  |  |  |  |  | 10 |  |  |  |  |  |  |  |
| | KB-1 |  |  |  |  |  |  |  |  |  | 4 |  |  | 1 |  | 3 |  | 1 |  |  |  |  | 1 |  |  |  |  |  |  | 3 |  |  |  |  |  |  | 2 |  |  |  |  |  | 10 |  | 1 |  |  |  |  |  |
| T. thermocopriae | JCM 7501 |  |  |  |  |  |  |  |  |  | 2 |  |  | 1 |  |  |  | 1 |  |  |  |  |  |  |  |  |  |  |  | 1 |  |  |  |  |  |  |  | 2 |  |  |  |  | 9 |  |  |  |  |  |  |  |
| | Ab9 |  |  | 1 |  |  |  |  |  |  | 3 | 1 |  | 1 |  |  |  | 1 |  |  |  |  |  |  | 1 |  |  |  |  | 1 | 1 |  |  |  |  |  | 2 |  |  |  |  |  | 11 |  |  |  |  |  |  |  |
| | A3 |  |  |  |  |  |  |  |  | 1 | 3 | 1 |  | 1 |  | 1 |  |  |  |  |  |  |  |  | 1 |  | 1 |  |  | 2 | 1 |  |  |  |  |  | 2 |  |  |  |  | 1 | 9 |  |  |  |  |  |  |  |
| T. pseudethanolicus | ATCC 33223 |  |  |  |  |  |  |  |  |  | 2 |  |  | 1 |  | 1 |  |  |  |  |  |  |  |  |  |  |  |  |  | 2 |  |  |  |  |  |  | 2 |  |  |  |  |  | 9 |  |  |  |  |  |  |  |
| | Ako-1 |  |  |  |  |  |  |  |  |  | 2 |  |  | 1 |  | 1 |  |  |  |  |  |  |  |  |  |  |  |  |  | 1 |  |  |  |  |  |  | 2 |  |  |  |  |  | 10 |  |  |  |  |  |  |  |
| | X514 |  |  |  |  |  |  |  |  |  | 2 |  |  | 1 |  | 2 |  |  |  |  |  |  |  |  |  |  |  |  |  | 1 |  |  |  |  |  |  | 2 |  |  |  |  |  | 10 |  |  |  |  |  |  |  |
| T. kivui | LKT-1 |  |  |  |  |  |  |  |  |  | 4 |  |  | 1 |  | 1 |  |  |  |  |  |  |  |  |  |  |  |  |  | 1 |  |  |  |  |  |  | 1 |  |  |  |  | 2 | 9 |  |  |  |  |  |  |  |
| T. uzonensis | DSM 18761 |  |  |  |  |  |  |  |  |  | 4 |  |  | 1 |  | 1 |  |  |  |  |  |  |  |  |  |  |  |  |  | 5 |  |  |  |  |  |  | 2 |  |  |  |  | 2 | 9 |  |  |  |  |  |  |  |
| | YS13 |  |  |  |  |  |  |  |  |  | 3 |  |  | 1 |  | 2 |  |  |  |  |  |  |  |  |  |  |  |  |  | 3 |  |  |  |  |  | 1 | 2 |  |  |  |  | 2 | 9 |  |  |  |  |  |  |  |
| T. ethanolicus | JW200 |  |  |  |  |  |  |  |  |  | 4 |  |  | 1 |  | 2 |  |  |  |  |  |  |  |  |  |  |  |  |  | 1 |  |  |  |  |  |  | 2 |  |  |  |  |  | 10 |  |  |  |  |  |  |  |
| | SR4 |  |  |  |  |  |  |  |  |  | 4 |  |  | 1 |  | 1 |  |  |  |  |  |  |  |  |  |  |  |  |  | 1 |  |  |  |  |  |  | 2 | 1 |  |  |  |  | 11 |  |  |  |  |  |  |  |
| | BSB-33 |  |  |  |  | 1 |  |  |  |  | 4 |  |  | 1 |  | 2 |  |  |  |  |  |  |  |  |  |  |  |  |  | 1 |  |  |  |  |  |  | 2 | 1 |  |  |  |  | 10 |  |  |  |  |  |  |  |
| | WC1 |  |  |  |  |  |  |  |  |  | 4 | 1 |  | 1 |  | 1 |  |  |  |  |  |  |  |  | 1 |  |  |  |  | 1 | 1 |  |  |  |  |  | 2 | 1 |  |  |  |  | 11 |  |  |  |  |  |  |  |
| | DSM 569 |  |  |  |  |  |  |  |  |  | 4 |  |  | 1 |  | 1 |  |  |  |  |  |  |  |  |  |  |  |  |  | 1 |  |  |  |  |  |  | 2 | 1 |  |  |  |  | 8 | 1 |  |  |  |  |  |  |
| | RT8.B1 |  |  |  |  |  |  |  |  |  | 4 |  |  | 1 |  | 1 |  |  |  |  |  |  |  |  |  |  |  |  |  | 1 |  |  |  |  |  |  | 2 | 1 |  |  |  |  | 11 |  |  |  |  |  |  |  |
| Th. sp. PSU-2 | PSU-2 |  |  |  |  |  |  |  |  | 1 | 7 | 1 |  | 1 |  | 2 |  |  |  |  |  |  |  |  | 1 |  |  |  |  | 1 | 1 |  |  |  |  |  | 1 |  |  |  |  |  | 11 |  |  |  | 2 |  |  |  |
| | RKWS2 |  |  |  |  |  |  |  |  |  | 4 |  |  |  |  | 1 |  |  |  |  |  |  |  |  |  |  |  |  |  | 1 |  |  |  |  |  |  | 1 | 1 |  |  |  |  | 11 | 1 |  |  |  |  |  |  |
| Th. xylanolyticum | LX-11 |  |  |  |  |  |  |  |  |  | 7 | 1 |  | 1 |  | 3 |  |  |  |  |  | 1 |  |  | 1 |  |  |  |  | 2 | 1 |  |  |  |  |  | 1 |  |  |  |  |  | 9 |  |  |  |  |  |  |  |
| Th. butyriciformans | USBA-019 |  |  |  |  |  |  | 1 |  |  | 7 | 2 | 1 | 1 |  |  |  | 1 |  |  |  |  |  |  | 1 |  |  |  |  | 1 | 1 | 1 |  | 1 |  |  | 1 | 1 |  |  |  |  | 12 |  | 1 | 1 |  | 1 |  |  |
| Th. aotearoense | SCUT27 |  |  |  |  |  |  |  |  |  | 6 | 2 | 1 | 1 |  | 3 |  |  |  |  |  |  |  |  | 1 |  |  |  | 1 | 1 | 1 | 1 |  | 1 | 1 | 1 | 2 | 2 |  |  |  |  | 11 |  | 1 | 1 | 2 |  |  |  |
| | JW-SL-YS485 |  |  |  |  |  |  |  |  |  | 6 | 2 | 1 | 1 |  | 2 |  |  |  |  |  |  |  |  | 1 |  |  |  | 1 | 1 | 1 | 1 |  | 1 | 1 |  | 2 | 2 |  |  |  |  | 12 |  | 1 | 1 | 2 |  |  |  |
| Th. saccharolyticum | NTOU1 |  |  |  |  |  |  |  |  | 1 | 7 | 2 |  | 1 |  | 1 |  |  |  |  |  |  |  |  | 1 |  |  |  |  | 1 | 1 | 1 |  | 1 |  |  | 2 |  |  |  |  |  | 13 |  | 1 | 1 |  | 1 |  |  |
| Th. thermosaccharolyticum | DSM 571 |  |  |  |  |  |  |  |  |  | 6 | 2 |  | 1 |  |  |  |  |  |  |  |  |  |  | 2 | 1 |  |  |  | 1 | 2 | 1 |  | 1 |  |  | 2 |  |  |  |  |  | 14 |  | 1 | 1 |  |  |  |  |
| | GSU5 |  |  |  |  |  |  |  |  |  | 6 | 2 |  | 1 |  |  |  |  |  |  |  |  |  |  |  | 1 |  |  |  |  |  |  |  |  |  |  | 1 |  |  |  |  |  | 10 |  | 1 |  |  |  |  |  |
| | M5 |  |  |  |  |  |  | 1 |  |  | 5 | 2 |  | 2 |  |  |  |  |  |  |  |  |  |  |  | 2 |  |  |  | 1 |  | 1 | 1 | 1 |  |  | 1 |  |  |  |  |  | 11 |  | 1 | 1 |  | 1 |  |  |
| | SP-H2 |  |  |  |  |  |  |  |  |  | 6 | 1 | 1 | 2 |  |  |  |  |  |  |  |  |  |  |  | 2 |  |  |  |  |  |  |  |  |  | 1 | 1 |  |  |  |  |  | 12 |  | 1 |  |  |  |  |  |
| | TG57 |  |  |  |  |  |  |  |  |  | 6 | 1 |  | 2 |  | 1 |  |  |  |  |  |  |  |  |  |  |  |  |  |  |  |  |  |  |  | 1 | 1 |  |  |  |  |  | 11 |  |  |  |  |  |  |  |
| | M0795 |  |  |  |  |  |  |  |  |  | 6 | 1 |  | 2 |  |  |  |  |  |  |  |  |  |  |  |  |  |  |  |  |  |  |  |  |  |  | 1 | 1 |  |  |  |  | 9 |  |  |  |  |  |  |  |
| NG. sp. RBIITD | RBIITD |  |  |  | 1 |  | 1 |  |  |  | 10 | 2 |  | 1 |  | 2 |  |  |  |  |  |  |  |  | 1 |  |  |  |  |  | 1 |  |  |  |  |  | 2 |  |  |  |  | 1 | 9 |  | 1 |  |  |  |  |  |
| Ca. fijiensis | DSM 13641 |  |  |  |  |  | 1 |  |  | 1 | 8 | 1 |  |  | 1 |  |  | 1 |  |  |  |  |  |  | 2 |  |  | 1 |  | 1 | 3 |  |  |  |  |  | 2 |  |  |  |  |  | 4 |  |  |  | 2 |  | 1 |  |
| Ca. polysaccharolyticus | A05MB |  |  |  |  |  |  |  |  |  | 3 |  |  | 2 |  | 2 |  |  |  |  |  |  |  |  |  |  |  |  |  |  |  |  |  |  |  |  | 1 |  |  |  |  | 1 | 5 |  |  |  |  |  |  |  |
| Cm. adulescens | DSM 17918 |  |  |  |  |  |  |  |  | 1 | 7 |  |  | 3 | 1 |  |  | 1 |  |  |  |  |  |  |  |  |  |  |  |  |  |  | 1 |  |  |  | 1 |  |  |  |  |  | 7 |  |  |  |  |  | 2 |  |
