# Supplementary figures and images for: Whither the genus Caldicellulosiruptor and the order Thermoanaerobacterales: phylogeny, taxonomy, ecology, and phenotype
Source: Front Microbiol. 2023 Aug 3;14:1212538. doi: 10.3389/fmicb.2023.1212538 (PMC10434631; doi:10.3389/fmicb.2023.1212538)

# Caldicellulosiruptoraceae Average Amino Acid Identities for CAZysome and Whole-Genome

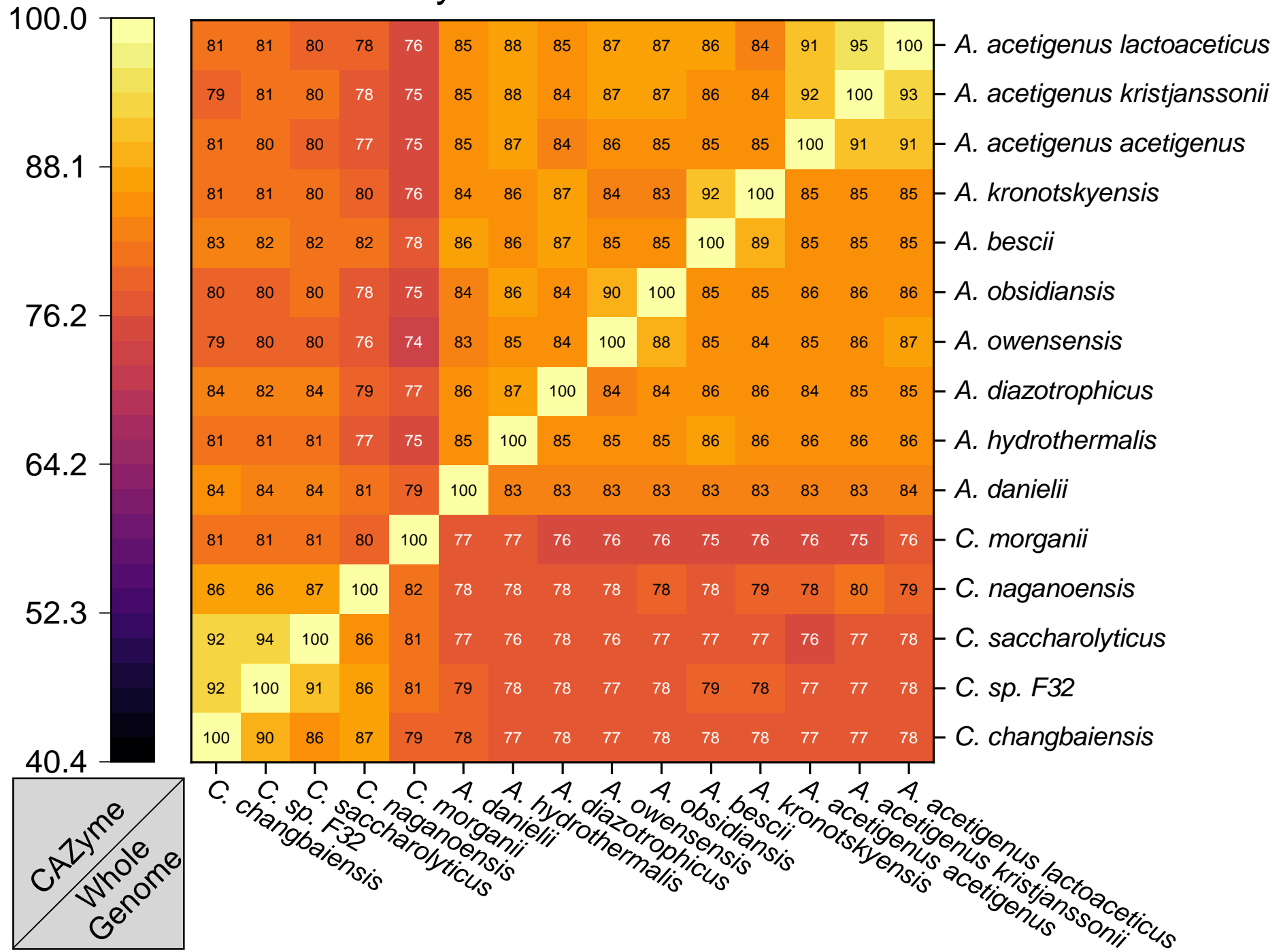

Supplement: Supplementary file 5 [file Image_3.PDF]
