# Supplementary material for: Whither the genus Caldicellulosiruptor and the order Thermoanaerobacterales: phylogeny, taxonomy, ecology, and phenotype
Source: Front Microbiol. 2023 Aug 3;14:1212538. doi: 10.3389/fmicb.2023.1212538 (PMC10434631; doi:10.3389/fmicb.2023.1212538)
Supplement: Supplementary file 6 [file Presentation_1.pdf]

Supplementary Material

Whither the Genus *Caldicellulosiruptor* and the Order Thermoanaerobacterales: Phylogeny, Taxonomy, Ecology, and Phenotype

Ryan G. Bing[1], Daniel J. Willard[1], James R. Crosby[1], Michael W.W. Adams[2], Robert M. Kelly[1*]

*** Correspondence:** Robert M. Kelly: rmkelly@ncsu.edu

1 Supplementary Data

Data File S1. ZIP file with output data from dbCAN, prior to curation.

Data File S2. ZIP file with clustered pangenome matrices from GET_HOMOLOGUES

2 Supplementary Figures and Tables

2.1 Supplementary Tables

Tables provided as Excel documents.

Table S1: Thermoanaerobacterales strains and assembly accession numbers obtained from NCBI

Table S2: Geographic location of *Anaerocellum* and *Caldicellulosiruptor* strains with genome sequences

Table S3: Geographic locations of Multi-Omics and Community Analyses indicating presence of Caldicellulosiruptorales

Table S4. Detailed list of CAZymes in the core and soft core CAZysome of the Caldicellulosiruptorales

Table S5: Proposed taxonomic reclassification of currently assigned Thermoanaerobacterales

Table S6. BLAST+ Average nucleotide identity data from pyani

Table S7. Average amino acid identity data from GET_HOMOLOGUES

Table S8. 16S ribosomal RNA identity data from Clustal Omega

Table S9. Curated dbCAN output

2.2 Supplementary Figures

Figures provided as PDF documents.

Figure S1. Pan, core, and softcore genome assessment of genera and families within the new Caldicellulosiruptorales and Thermoanaerobacterales

Figure S2. CAZyme summary (not including GHs) for the Caldicellulosiruptorales and Thermoanaerobacterales.

Figure S3. Caldicellulosiruptoraceae Average Amino Acid Identities for the CAZysome and Whole-Genome
